# LIDB: A multi-omics database of *Lagerstroemia indica*

**DOI:** 10.6026/973206300222316

**Published:** 2026-04-30

**Authors:** Lei Wang, Bai Li, Gaojun Chong

**Affiliations:** 1 Department of Biotechnology, Jiaxing Academy of Agricultural Sciences, Jiaxing, Zhejiang, China, 314016

**Keywords:** *Lagerstroemia indica*, multi-omics, database

## Abstract

The *Lagerstroemia indica* (Crepe Myrtle) is an economically important ornamental and medicinal plant, with significant potential for functional genomics and breeding. However, there is no comprehensive, centralized database integrating multi-omics data. Therefore, it is of interest to the development of the *Lagerstroemia indica* MultiOmics Database (LIDB), a web-based platform designed to integrate and analyze genomic, transcriptomic and proteomic data. The LIDB is a valuable resource for researchers, breeders and biotechnologists, facilitating data-driven discoveries in *Lagerstroemia indica* genomics.

## Background:

*Lagerstroemia indica* is a widely cultivated ornamental plant, native to Southeast Asia and has been extensively used in landscaping, as well as in traditional medicine [1]. Various promising omics technologies have emerged in the past few decades, these omics-based approaches have proven to be valuable for exploring the genetic and molecular basis of crop development by modifying DNA, transcript levels, proteins, metabolites and mineral nutrition in the context of environmental and physiological stress responses [2]. Several omics approaches, such as genomics, transcriptomics, proteomics, metabolomics and phenomics, have revealed each corresponding molecular biological aspect integrated with plant systems [3, 4]. The advent of next-generation sequencing (NGS) technology has brought about high-throughput and rapid data generation of genomes, transcriptomes, proteomes, metabolomes and phenomes [5]. The integration of multiple omics approaches can elucidate gene functions and networks under physiological and environmental stress conditions. Comprehensive multi-omics approaches with powerful technologies have been used to identify and decipher the essential components of stress response, senescence and yield in various important economic crops [6]. These omics approaches have been implemented in several important crops, including wheat, soybean, tomato, corn, millet, cotton and other economic crops [7]. However, there are relatively few reports on omics studies in *Lagerstroemia indica*. Given the growing need for centralized resources that enable easy access to genomic and multi-omics data. Therefore, it is of interest to describe the development of the *Lagerstroemia indica* Multi-Omics Database (LIDB).

## Materials and Methods:

The main procedures of *Lagerstroemia indica* Multi-Omics Database (LIDB) construction are illustrated in Figure 1 (see PDF), including data collection, data integration. Regarding the database, we have extracted most of the related genome and transcriptome data from NCBI Gene Expression Omnibus. Data manually retrieved from the literature account for a minor portion of the total multi-omics data, which was obtained by querying PubMed using keywords and handpicking several essential findings from recent years. We curated and annotated the collected data with the help of publicly available knowledge. For the annotation of genes, we considered such databases as Gene [8] as well as R packages include biomaRt [9] and clusterProfiler [10]. Multi-omics integration analysis involves systematic data processing and modeling across genomic, transcriptomic, proteomic and metabolomic datasets. The workflow begins with quality control using platform- specific tools (FastQC for sequencing data, MaxQuant for proteomics and XCMS for metabolomics) followed by batch effect correction via ComBat [11]. Data normalization employs Z-score transformation to enable cross-omics comparison. For pairwise integration (*et al.*, transcriptome-proteome), Spearman correlation identifies concordant molecules, while sparse canonical correlation analysis (sCCA) reveals co-varying modules between metabolomic and microbiome data [12]. Higher-order integration utilizes MOFA+ for dimensionality reduction to extract latent factors shared across omics layers [13]. Machine learning approaches like MOVICS [14] integrate NMF and consensus clustering for patient stratification [15]. Pathway mapping is performed using MetaboAnalyst with KEGG enrichment (FDR <0.05) [16].

## Database and data statistics:

In the current version, LIDB documents 97 manual processed datasets of *Lagerstroemia indica*. Each entry is associated with the PMID and gene symbol. Meanwhile, we also collected 6 other economic crops for users to use as the reference. LIDB provides a user-friendly interface with interactive plots and searching bars in each module. In the 'Search' page. Users can retrieve data by location, gene symbol or keywords to retrieve related data. LIDB provides brief description of search results in the 'Search Result' page. To gain more detail information of a specific entry, users can click the 'Details' button. Additional information such as PubMed ID, GO annotations are displayed in the single-record page. It also integrates multi-omics data into the genome browser, JBrowse, that provides reference sequence, annotation, population statistics and genome-wide-associated analysis results for agronomic traits.

The tool page offers a plethora of bioinformatics tools, including Local Alignment Search Tool (BLAST), GWAS Single-Trait for laboratory research. The database was constructed based on LAMP (Linux, Apache, MySQL and PHP) mode. MySQL was selected to store biomarker information. The front end of the webpage was written in HTML, CSS and JavaScript, while PHP was used to realize the interaction of the webpage. The database was running under the environment of Linux and Apache. The website is freely available to all without the need for registration or login. Designed for cross-platform compatibility, it ensures a user-friendly interface across computers and tablets for broad accessibility.

## Utility:

*Lagerstroemia indica* is a representative species of the genus *Lagerstroemia*. The extensive application of multi-omics technology in *Lagerstroemia* research has enabled people to better understand many unique genetic bases and origin histories of *Lagerstroemia*, providing important information for a more in-depth, comprehensive and systematic understanding of *Lagerstroemia* and providing a broader space for the innovation and quality improvement of germplasm resources in the future, but there are still some limitations in the research process. First, although genomic studies of *Lagerstroemia* have taken a leading role in multi-omics research, the available chromosome-level genomes are still relatively limited in both size and diversity of studied accessions, especially when compared with well-established crop systems such as rice or maize. Moreover, several advanced high-throughput sequencing technologies-such as spatial transcriptomics and single-cell omics-have not yet been applied to *Lagerstroemia* and the use of transcriptomic, metabolomic, proteomic and integrated multi-omics analyses remains relatively scarce. Nevertheless, with the continuous accumulation of data and the emergence of more powerful integration methodologies, we anticipate that resources such as the *Lagerstroemia indica* Database (LIDB) will become increasingly valuable tools for researchers in this field.

## Future work:

As the database continues to evolve, further integration with additional omics layers, such as epigenomics and sc-RNA-seq, will expand its capability to provide a more holistic view of plant biology. The expansion of data through collaborative efforts and the incorporation of community-driven contributions will ensure that LIDB remains at the forefront of *Lagerstroemia indica* research, helping to foster innovation in both fundamental biology and practical applications.

## Funding:

This research was funded by the Research on Radiation Breeding and Tissue Culture Technology of *Lagerstroemia indica*, grantt number 2021C02071-4-5.

## Availability:

LIDB can be visited freely via http://lidb.org.cn.
